# Supplementary material for: Ergonomic Design and Performance Evaluation of H-Suit for Human Walking
Source: Micromachines (Basel). 2022 May 25;13(6):825. doi: 10.3390/mi13060825 (PMC9227600; doi:10.3390/mi13060825)
Supplement: Supplementary file 1 [file micromachines-13-00825-s001.zip › micromachines-1699335-supplementary.pdf]

micromachines

Supplementary material to article

Ergonomic Design and Performance Evaluation of H-Suit for Human Walking

Leiyu Zhang [1], Zhenxing Jiao [1], Yandong He [1] and Peng Su [2,*]

[1] Beijing Key Laboratory of Advanced Manufacturing Technology, Beijing University of Technology, Beijing 100124, China; zhangleiyu@bjut.edu.cn (L.Z.); jiaozhenxing0109@163.com (Z.J.); heyandong13@163.com (Y.H.)
[2] School of Electromechanical Engineering, Beijing Information Science and Technology University, Beijing 100192, China
* Correspondence: supeng@bistu.edu.cn; Tel.: +86-010-18911027599

Supplementary material

Supplementary Table S1. Parameters of the lower limb.

Parameter	Number
Hip angle θ/°	−20~30
Distance between hip and knee l_{hk}/mm	400
Thigh diameter d/mm	160
Waist thickness T_w/mm	240
